# A Resume of the Different Preparations of Gold Used for Filling Teeth

**Published:** 1884-02

**Authors:** George C. Brown


ARTICLE III.

### A RESUME OF THE DIFFERENT PREPARATIONS OF GOLD USED FOR FILLING TEETH.

BY DR. GEORGE C. BROWN.

It is not known when, where or by whom the operation of filling teeth was introduced. It has been claimed to go back before the Christian era, but no definite account has ever been handed down to us of the ancient methods of filling teeth. The first authentic writings on the subject may be found in a work published by John Hunter, F. R. S., London, 1778. Under the heading of "Stopping of the Teeth," he says:

"If the destruction of the life of the tooth, either by drawing and restoring it again, or by actual or potential cauteries, has not been effected, and only the cure of the inflammation has been attempted, another method of preventing inflammation is to be followed, which is to allow as little stimulus to take place as possible. The cavity of the tooth not being capable of taking this alarm, like most other cavities in the body, and, of course, not suppurating, often no more is necessary either to prevent the inflammation from taking place altogether, or extending further, than to exclude all extraneous irritating matter; therefore, the stopping up the cavity becomes in many places the means of preventing future attacks of the inflammation, and often retards even the progress of the disease, that is, the further decay of the teeth, so that many people go on for years thus afflicted. But it is a method which must be put in practice early, otherwise it cannot be continued long, for if the disease has done considerable damage to the inside of the tooth, so as to have weakened it much, the whole body of the tooth most probably will soon give way in mastication; therefore, under such circumstances, the patient must be cautioned not to make too free with his tooth in eating.

" Gold and lead are the metals generally made use of for stopping teeth. Gold being less pliable, must be used in the leaf. Lead is so soft in any form as to take on any shape by a very small force. Stuffing the hollow tooth with wax, galbanum, etc., can be but of very little service, as it is in most cases impossible to confine these substances, or preserve them from being worn away. However, they have their uses, as it is a practice which the patients themselves can safely put in execution.

" It often happens from neglect, and much oftener in spite of all the means that can be used, that the tooth becomes so hollow as to give way, whereby the passage becomes too large to keep in any of the above mentioned substances; however, in this case, it sometimes happens that a considerable part of the body of the tooth will still stand, and then a small hole may be drilled through this part, and after the cavity hath been well stopped, a small peg may be put into the hole, so as to keep in the lead, gold, etc. But when this cannot be done, we may consider the broken tooth as entirely useless, or at least it will soon be so, and it is now open to attacks of inflammation, which the patient must either bear, or submit to have the tooth pulled out. If the first be chosen, and the repeated inflammations submitted to, a cure will be performed in time, by the stump becoming totally dead."

In the work of Joseph Fox, F. R. S., published in the year 1806, under the head of " Stopping the Teeth," he says:

" In stopping the teeth, the first thing to be done is to cleanse the cavity from all extraneous matter, and to wipe it out dry; then a piece of gold or tin foil leaf is to be introduced and carefully and firmly pressed in, so as to completely fill up the cavity, and allow the mouth to be closed without forcibly pressing upon it. The stopping is then to be polished, and being quite smooth, it will not be in any way offensive to the tongue. A decay in the central part of the teeth is the most favorable situation for retaining the stopping; when it is in the side, or between the teeth, the pressure of the food is liable to displace it, and therefore it requires frequent renewals."

Of instruments for stopping the teeth, he says:

"They consist of a hook for picking extraneous substance out of the cavity; a straight and a curved instrument for pushing the stopping into the tooth, and an instrument with a bulbous-formed end, to be used as a burnisher in polishing the surface of the stopping."

Harris says that it was not until the year 1800 that the use of gold became common among dentists. For the next twenty or thirty years the use of gold was confined mostly to dentists in the large cities. Dr. Eleazer Parmly states that the first gold filling he ever saw was in 1815 and this was put in by Dr. Waite, of London.

About the years 1812–13 Marcus Bull, of Hartford, Conn., began the gold-beating business. He afterward moved to Philadelphia, and in the year 1818, in connection with Chas. Abbey, who had been his apprentice, began regularly the manufacture of gold foil, he being the first manufacturer of gold-foil in this country, venturing at first only to make an ounce or two at the time, the numbers running over 4, 12, 16. From that time there has been a gradual advance in the amount manufactured. Gold was first used in the form of pellets placed in the cavity and forced by wedge-shaped instruments against the sides, finishing with a centre-piece forced into a wedge-shaped aperature. The first notice I have been able to discover of crystallized gold in connection with our profession, is in an article by Dr. C. T. Jackson. It is entitled, "A New Method of Extracting Pure Gold from Alloys and from Ores. By C. T. Jackson U. S. G. S." He says: "I have made several useful applications of the gold sponge thus prepared, and had a tooth plugged with it in October, 1846, to which purpose it is well adapted. By moderate pressure, the spongy gold becomes a solid mass, and burnishes quite brilliantly." We hear nothing further of crystal gold until 1853, when Mr. A. J.

Watts, of Utica, New York, and Mr. Joseph Barling, Maidstone, Kent, England, appeared in the field almost at the same time. The nature of this invention consists in dissolving out the mercury by nitric acid, and then subjecting the new conditioned, but as yet unfinished, gold to the action of a particular heat, whereby it is rendered coherent, soft, and malleable, thus fitting it for the purpose of filling teeth. In less than a year this material was manufactured and advertised by at least three different parties—the original patentee, under the name of A. J. Watts & Co., and N. P. & H. R. White, both firms being located at Utica, New York, and Messrs. Taft and Watts, of Xenia, Ohio. As a matter of curiosity, I will copy their advertisements:

"Watts' Patent Prepared Gold for Filling Teeth. This is a preparation in various forms, of a spongy, laminated, or crystalline character, so soft, plastic, and adhesive as to admit of being welded, piece by piece, by simple pressure, into a body so solid that it may at once be beaten into foil or drawn into wire. It possesses the following advantages over foil: 1. It must necessarily be pure gold. 2. It may with ease, and with comparatively unpracticed hands, be worked up to any external form in the tooth, with a perfection and beauty which it is believed impossible to attain with foil in the hands of the most skillful. 3. It adheres to the tooth with such tenacity that it can only be extracted by drilling or breaking the tooth, and when thus removed the plug will be found covered with numerous small particles of bone adhering to it. 4. It requires for its use less practice and skill to accomplish good work, while it amply repays the skillful operator in the production of splendid fillings. 5. It makes an absolutely solid plug."

These preparations were soon followed by "Morgan's Plastic Gold," Lamm's Shredded, etc. Sponge gold at once took a high stand and was heralded far and wide. It had the approval of such men as Dwinell and Arthur, and was written about in all the journals, discussed at the meetings, and for a time was a great hobby with many. It was somewhat amusing to hear the claims put forward by some of its friends. One point was, that it would work as well under water as dry; another claimed that he never took the trouble to dry out the cavities when using it; he rather preferred to have them filled with saliva, as it made the gold work better; and one gentleman said he had worked it in flour with perfect success. For a time it was extensively used by a large number of the profession. I used it with very good results, but gradually the profession fell back to foil. In the year 1855, at the meeting of the American Society of Dental Surgeons, Dr. J. S. Clark, of New Orleans, described his method of introducing gold in the shape of cylinders:

"The foil is carefully rolled into cylinders, as a bolt of cloth is rolled, and of a length to suit the depth of cavity, and intended to be a little longer than the cavity is deep. They are rolled of all sizes, from a full sheet down to the size of cambric needle wire. Some are made conical in shape, but most are plain, straight cylinders. Some are rolled very lightly, others quite hard. The application is to place soft (or lightly rolled) ones in the cavity endwise, being careful that each cylinder reaches the bottom of the cavity and protrudes a little outside the orifice. When the cavity is apparently full, a round instrument is passed down between them, another cylinder (a little harder rolled), is forced up. This is repeated, decreasing in size of instrument and cylinder (the latter of increased hardness), until no more can be introduced."

In the same year Dr. Arthur, of Baltimore, brought before the profession a new method of using *gold-foil*. It consisted in thoroughly annealing the foil in a spirit lamp before using, thereby developing its cohesive properties: "There are two methods in which gold may be prepared in the manner proposed. It may either be used in the form of pieces cut from a rope, or the sheet may be cut up or torn into small pieces, without folding or rolling it up."

With the advent of crystal gold a new field was opened to the profession, in the building up of lost portions and the restoring of gold crowns to the badly broken and decayed teeth. With cohesive foil it was carried still further, and was followed by what was known as contour filling, a style of filling now brought to a great state of perfection. Operations not thought of before were now of every-day occurrence, and this new department gave operative dentistry a great start forward.

A few years after Dr. Atkinson introduced to the profession that delicate little instrument known as the mallet, for condensing gold, Then arose another great and important question, what weight was best adapted for condensing the gold? One said one ounce; another two and three; another half a pound. Some wanted steel, some wood, some lead, etc. Never shall I forget calling upon one of the distinguished members of the profession, and during my brief stay asking him to show me the kind of mallet he used and liked best. He said certainly, and went to a stand near by and brought out at least a peck, and said, "There are the kinds I use." What did I behold? Mallets in every form and weight, from the tiny toy to a sledge-hammer; some in all their original form and beauty, others beaten out of all form and comeliness, and as I gazed on the battered remains before me and my mind wandered back to the time when this transition was going on, and I wondered to myself how many lived to tell the tale of their escape from these battles with mallets.

Immediately following the mallets came the heavy gold fever. Up, up, it went, from the old 4, 5, 6, to 30, 60, 120, 240, and still higher, until some said a perfect filling could hardly be put in unless you used 480.

After heavy gold settled a little, came the different forms of gold. Who among the older members does not remember the oily-tongued French gentleman and his tooth-brush handle. He was like a shadow at our meetings; in fact, he was ever present with us, just to show how easy it was to fill a cavity if you only used his gold-foil cylinders and *blocks*; just so easy, and he would demonstrate to you with that everlasting tooth-brush handle, and even if the filling got wet, "no harm; shust so good as before; you shust burnish the surface and my gold blocks stick shust so well as before, you see."

To ascertain as nearly as possible the numbers and kinds of gold most used by the profession at this time, I addressed a letter to a number of the leading manufacturers of gold-foil, asking them the following questions: 1. How many kinds of gold do you manufacture? 2. Which has the largest sale, soft or cohesive? 3. What number is most used? From Charles Abbey, the oldest manufacturer in the country, I received the following: "We do not manufacture anything but gold foil. No. 4 with us commands the largest sale. Soft or old-fashioned (with us a specialty), and cohesive, but made from pure gold. The sale of soft and cohesive is nearly three to one; that is, we sell three ounces of soft gold to one of cohesive. There are dentists dealing with us who have never even tried cohesive gold, and to whom the very name is offensive." R. S. Williams says: "I make in gold for filling about one hundred and twenty-five different preparations, which include different sizes, shapes, qualities, etc., to which may be added a large number of special sizes, made for individuals, and not advertised. Probably the whole number would be about two hundred." Their report as to the kind most used is soft foil No. 4. James H. Ashmead & Son, give the following: "We manufacture to kinds of gold foil, namely, soft and cohesiva. No. 4 is used largely in excess of others, and soft foil is the most used. During the past fifteen years great improvements have been made in the process of refining gold, which we have taken advantage of, and all gold which goes out of our establishment is absolutely pure and of uniform working qualities, We give our personal attention to refining all gold used for foil. The want of time forbids giving a complete sketch of the manufacture of gold-foil, but we will give you a few ideas: First, we obtain the gold principally in two forms, United States bar or coin After going through the process of refining, the gold is melted in sand crucibles and cast in ingots about one inch wide, which are then rolled down to the desired thinness, according to the number of foil to be made. The ribbon thus obtained is rolled up and weighed for what is termed a 'beating,' which, after being annealed, is given to the workman, who, with a pair of dividers marks it into a certain number of pieces; then cuts the same up with shears, each piece being about an inch and one-eighth square, which when beaten out makes a leaf of foil. The process of beating, of course, renders the gold hard, and each sheet has to be annealed. A most important part of the business is to have each sheet receive the exact degree of heat. It is then trimmed, weighed up, booked, etc., ready for the market."

Edward Rowan says: "We manufacture two kinds of gold—rolled gold and beaten gold—and four varieties of the beaten gold, viz.: unannealed, cohesive, medium and soft: also a variety of gold rolls. There is more soft than cohesive foil used, and there is a much larger sale of No. 4 than of any other number."

Thus you will see history repeats itself. You will recollect that at first all low numbers was made; since then, we have had numbers extendiug as high as 480. We have had the sponge and crystal gold preparations. Cohesive gold had its run and was gradually laid aside, until now we come back almost to where we started from. You cannot but see from the letters just read that soft foil No. 4 is to-day more largely used than any other preparation of gold.

My experience is that with any form of gold, be it rolled or beaten, cylinders or blocks, foil or sponge, heavy or light, all alike require skill, patience and care. It also requires labor, practice and experience to properly work it. It requires an educated brain, guiding will and disciplined muscles to use it successfully. The student must often bear many crosses before his eyes behold the glittering crown. It requires not only time to learn to use it, but time to use it properly. None of them can be slighted with impunity. —*Transactions of New York State Dental Association.*

